# Inter Spinal Fixation and Stabilization Device for Lumbar Radiculopathy and Back Pain

**DOI:** 10.7759/cureus.19956

**Published:** 2021-11-28

**Authors:** Soubrata V Raikar, Arun A Patil, Deepak K Pandey, Sidharta R Kumar

**Affiliations:** 1 Pain Management, Midwest Anesthesia Pain Management, Elkhorn, USA; 2 Surgery, Creighton University School of Medicine, Omaha, USA; 3 Medical Physics, Less Exposure Surgery (LES) Institute, Malden, USA

**Keywords:** isp, ifd, inter-spinous fusion device, inter-laminar fixation, inter-spinous fixation device, low back pain, lumbar radiculopathy, inter-spinal distraction fixation, lumbar disc disease, spinal stenosis

## Abstract

Introduction: Generally, interspinal distractor fixation devices are used for severe low back pain associated with neurogenic claudication, and radiculopathy with central or lateral recess stenosis and/or foraminal narrowing. In this paper, the authors result in cases of severe low back pain and lumbar radiculopathy in whom this device was used with excellent results.

Method: This is a retrospective study. Patients were contacted via phone call and their pain score and other data were recorded at different timelines. The final data presented in this paper are the data collected at the final follow-up that ranges from 14 months to 24 months. Surgeries were performed in the outpatient setting and although no identifiable patient information is included in this paper, yet, patients were asked for their verbal consent. The patient data are only included if verbal consent was obtained.

Results: Over the past 24 months, 13 patients with disc protrusion and/or central and/or foraminal spinal stenosis were treated with this procedure. Follow-up ranges from 14 months to 24 months with a median of 19 months, male/female ratio of 6/7, and a median age of 68 years. There were no complications or reoperation. Statistical analysis showed significant improvement in the Numeric Pain Rating Scale (NPRS) for back and radicular leg pain (p-value = 0.000552 for back pain and p-value = 0.000291 for radicular leg pain).

Conclusion: The system reported in this paper is a solid fixation system that works both as a distractor and internal decompressor of the spinal canal. It is simple to use and safe. Though the number of patients is small, statistically significant improvement was reported at a median follow-up of 19 months.

## Introduction

The degenerative process of the spine can result in disc disease, spinal stenosis, facet arthropathy, and foraminal narrowing. Disc disease can result in radicular signs and symptoms; spinal stenosis can cause neural claudication, radicular symptoms, motor function loss; facet arthropathy can cause joint pain, and foraminal narrowing can cause radicular signs and symptoms. Traditionally, laminectomy, foraminotomy, discectomy, facet screws fixation, and fusion are the surgical procedures performed for these conditions. The interspinal distraction (ISD) or spacer devices were invented as distracting the spinous process widens the foramen, decreases the pressure on the disc and facets, and opens the spinal canal by stretching the ligamentum flavum. Though initial results on these devices were encouraging some studies cast doubts on their effectiveness [1-3]. More recently interspinal distraction with dynamic stabilization (ISDDS) and interspinal distraction with rigid fixation (ISDRF) devices were invented. These devices show promising results [2,4-12] and are approved by the U.S. Food and Drug Administration (FDA) for single-level fixation. In this paper, the authors present their preliminary study with Inspan device using a retrospective study.

## Materials and methods

Over the last 24 months, the authors have used the Inspan device (Inspan, LLC Malden, MA, USA) in 13 patients. The male-female ratios are 6/7; the age range is from 39 to 86 years with a median of 68 years. The following study covers 14 months to 24 months with a median of 19 months follow-up. All patients had a history of chronic pain syndrome and had extensive pain management treatment including epidural steroids injection and physical therapy. All patients had low back pain with shooting radiculopathy. Those with significant stenosis also had neurogenic claudication. Seven patients had moderate to severe or severe spinal stenosis, others had disc protrusion and/or foremen narrowing, one patient had grade 1 listhesis. Patients’ back pain and radicular leg pain were scored pre and post-operatively using the numeric pain rating scale (NPRS).

Description of the system

**Figure 1:**
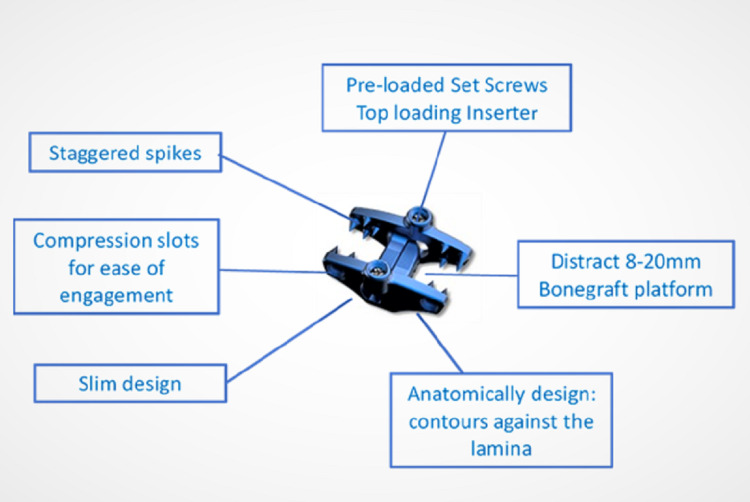
Inspan - interspinous distraction/fusion device ISP: interspinous plate, ISD: interspinal distraction, ISF: interspinal fusion

The device is an interspinous distraction fixation device (Figure 1), is low profile, and is made of medical-grade titanium, Ti6AL-4VELI. The two plates (also called wings) have aggressive spikes strategically positioned on them for strong fixation without causing fracture of the spinous processes. It has expansion and compression capability that allow the distraction and the compression of the affected spinal segment during the surgical procedure. It also has a dual interlocking hub with dual set screws providing added fixation. It is designed to bottom out on the lamina to provide maximum fixation and distraction.

Operative procedure

**Figure 2:**
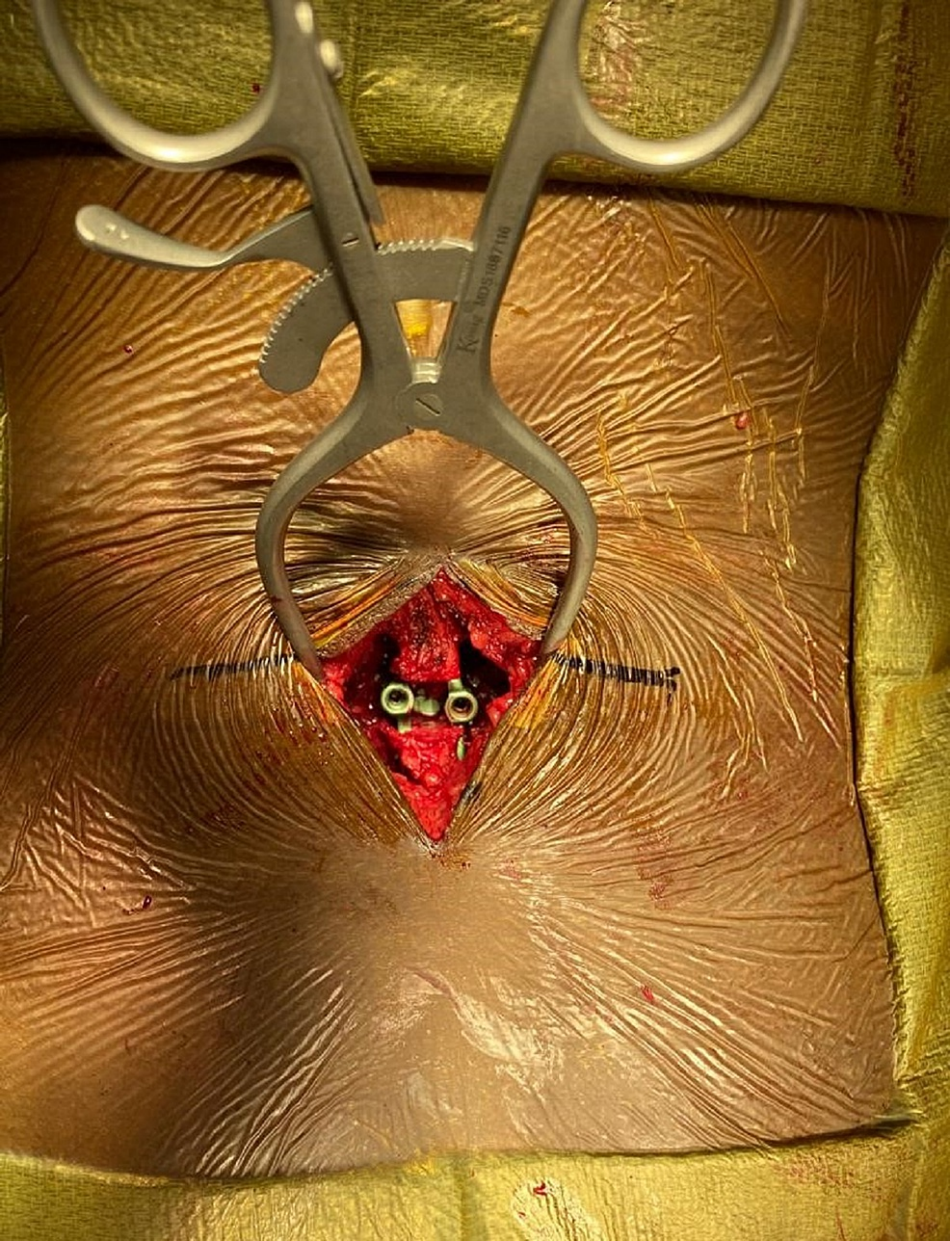
Intra-Op image of incision for implanting Inspan ISP device and the inserted Inspan

The procedures were done under general anesthesia or local anesthesia with heavy sedation in the prone position. Intra-operative fluoroscopy was used, as needed. A small incision 3.5-4 cm in length was made (Figure 2).

**Figure 3:**
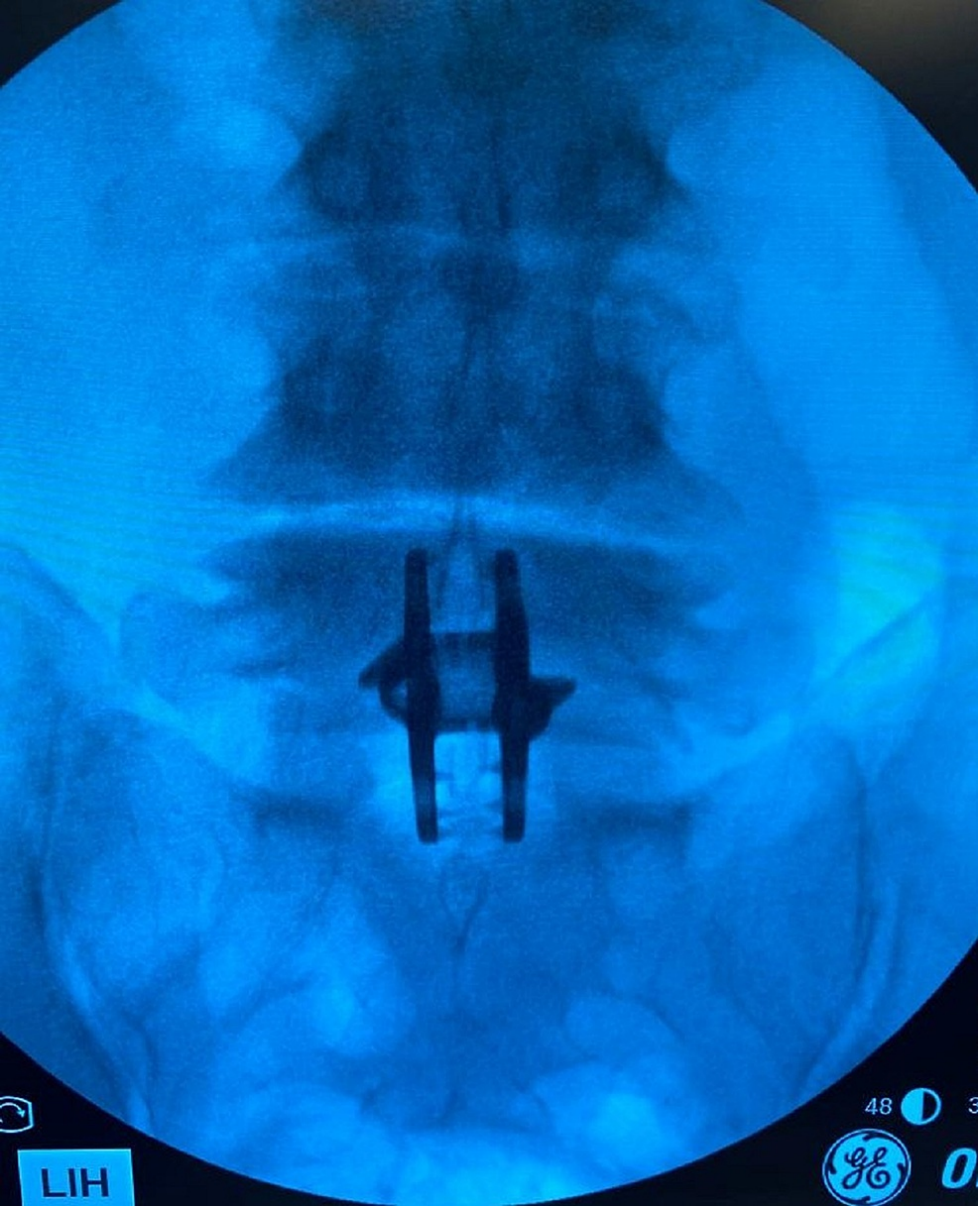
Anterior-posterior X-ray image of the inserted Inspan ISP construct at L4-L5 level ISP: interspinous plate, ISD: interspinous device

**Figure 4:**
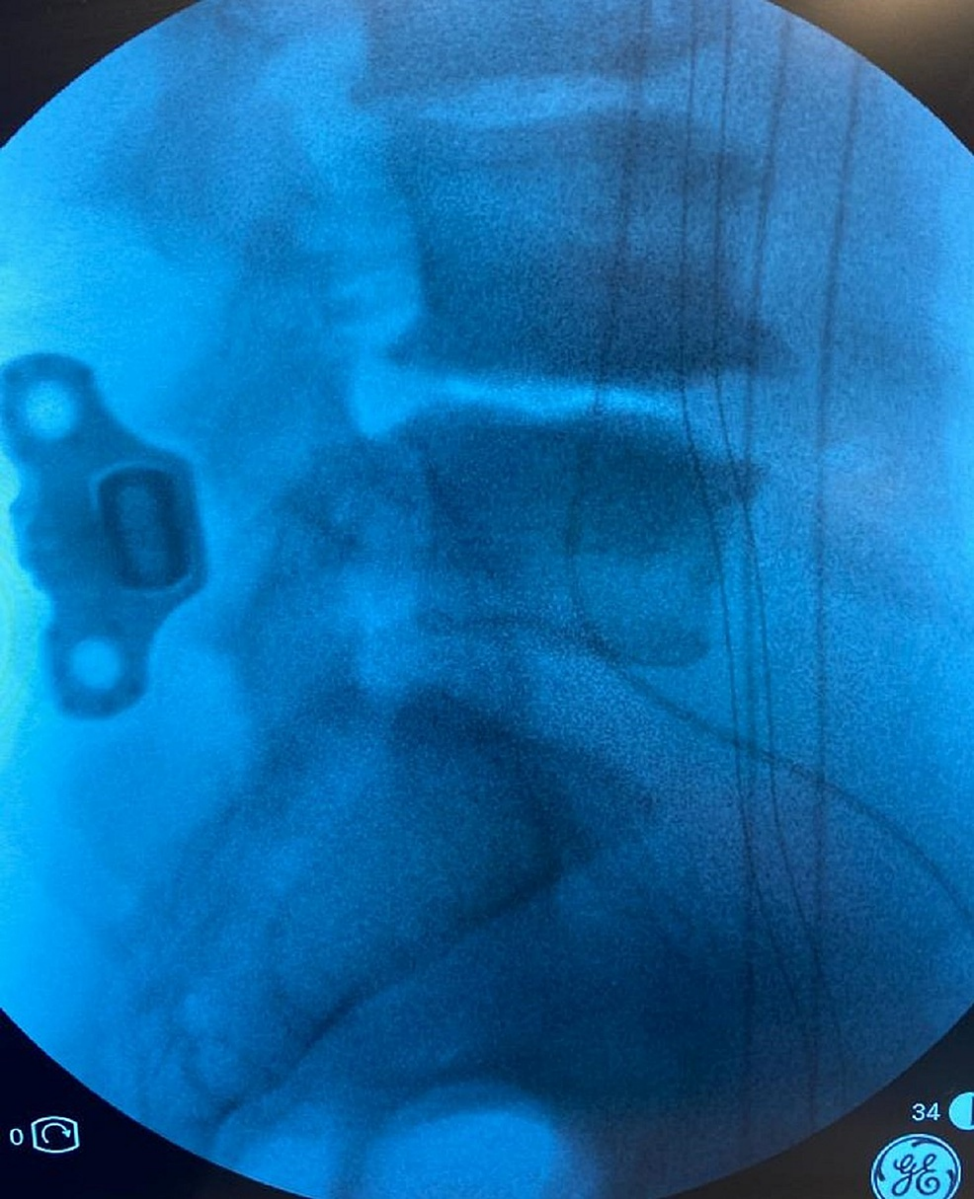
Lateral view of the inserted Inspan construct at L4-L5 level ISP: interspinous plate, ISD: interspinous device

The para-laminar muscles were separated from the lamina and retracted laterally. The interspinous ligament was then removed deep down to the ligamentum flavum. Bony margins of the spinous processes in the interspace were roughened up. Distraction distance was measured using a temporary distractor under fluoroscopic control. Distraction was increased until the posterior disc space increased by 1.5 mm in height. An appropriate size device was then inserted and hammered down to the full depth (Figures 3 and 4).

Fluoroscopic images were obtained to confirm the intended distraction. The wings of the inspan interspinous distractor were then compressed into the spinous processes, and the system was locked in position. Then allograft bone putty was placed between the roughened surfaces of the spinous processes. The wound was then closed.

Statistical analysis

A sample of 13 patients was analyzed for pre-op and post-op back pain as well as radicular leg pain. The data were recorded using the NPRS, and data gathering was conducted for the back pain and radicular leg pain. Analysis for back and leg pain reduction was done via “t-test: paired two samples for mean” and the results were considered significant for p-value < 0.05. Statistical analysis was conducted via the Microsoft Excel data analysis tool.

## Results

A sample of 13 patients was analyzed for pre-op and post-op back pain as well as leg pain. The data (Table 1) were recorded using the NPRS and the analysis was conducted for the back pain and leg pain. Analysis for back and leg pain reduction was done via “t-test: paired two samples for mean” and the results were considered significant for p-value < 0.05. Average back pain reduction for the sample was recorded as 3.27 at NPRS, which was statistically significant (t12 = 4.26, p-value = 0.00055). Average radicular leg pain reduction for the sample was recorded as 1.38 at NPRS, which was statistically significant (t12 = 3.23, p-value = 0.0035). “t-test: two-sample assuming unequal variance” analysis was conducted to see the effectiveness of the surgery for two different age groups and female versus male. Study of back and leg pain reduction between age group ≥68 years (7) versus <68 years (6) with t11 = 1.849, p-value = 0.0.046 for back pain and t11 = 1.863, p-value = 0.045 for leg pain indicates that there was asymmetry in pain reduction in two age groups; however, this was amplified as patient# 8 had an increase in pain score (from 5 to 8). Further evaluation of the imaging reports of patient #8 revealed that this 86-year patient had degenerative changes to multiple disc levels with evidence of scoliosis above and below the operating level of L4-L5, which may be causing the increase in pain.

**Table 1:** Data showing patient demographic, diagnosis, MRI findings, treatment, follow-up time, pre-op/post-op low back, and leg pain L3: lumbar level 3, L4: lumbar level 4, L5: lumbar level 5, S1: sacrum level 1

Patient#	Age (years)	Sex (M/F)	History	MRI findings	Operation	Follow-up (months)	Pre-Op/Post-Op NPRS for low back pain	Pre-Op/post-Op NPRS for leg pain
1	85	F	6 years of right hip pain and difficulty walking due to pain. Tried brace, spinal cord stimulator, and epidural steroid injections.	L3-4 right disc protrusion	Inspan at L3-4 with posterior fusion	24	7/1	9/6
2	60	M	Sharp burning pain in the left leg for several years. Previous laminotomy at L4-5.	Moderate to severe central canal stenosis L4-5	Inspan at L4-5 with posterior fusion	20	6.5/4	6.5/4
3	73	F	7 years of pain in her legs while walking. Tried epidural steroids.	L4-5 severe central canal stenosis	Inspan at L4-5	22	7/3.5	7/3.5
4	69	M	10 years of low back and right leg pain increased by standing, Has scoliosis. Tried epidural steroids.	L4-5 moderate central stenosis	Inspan at L4-5 with posterior fusion	19	9/6	9/6
5	67	F	4 months of the right knee and lower back pain	L3-4 right disc protrusion and grade one listhesis	Inspan at L3-4and posterior fusion	22	5/0	5/0
6	70	F	Left leg shooting pain for 4 years. Has scoliosis.	Mild central stenosis L4-5	Inspan at L4-5 and posterior fusion	21	8/8	7/0
7	68	F	Constant ache in both lower extremities for 18 months.	L3-4 severe central stenosis	Inspan at L3-4 and posterior fusion	17	6/2	6/2
8	86	M	Low back and left knee pain for 6 months, worse when he walks.	Moderate to severe central stenosis L4-5	Inspan at L4-5 and posterior fusions	17	5/8	5/8
9	69	F	Pain in both legs and knee for 6 months.	Mild bilateral lateral recess stenosis at L3-4.	Inspan at L3-4 and posterior fusion	17	7/6	7/6
10	39	F	Low back pain with pain in both legs, aching and sharp in nature, tried epidural steroids	Small disc protrusion at L5-S1 with mild bilateral foramen narrowing.	Inspan at L5-S1 with posterior fusion	19	7/3	7/0
11	52	F	Low back pain with sharpness in both legs and difficulty walking for 2 years.	Moderate to severe central stenosis with bilateral foramen narrowing at L3-4	Inspan at L3-4 and posterior fusion	19	9/2.5	9/0
12	46	M	Low back pain with radiation into both legs for 5. Tried epidural asteroids.	Bilateral L4-5 mild central stenosis with bilateral foramen narrowing	Inspan at L4-5 with posterior fusion	14	6/3	6/3
13	63	M	Low back pain with radiation into the right leg for 5 years. Recently started limping with numbness in the right leg.	Lateral recess stenosis L4-5 with severe narrowing of the foramen	Inspan at L4-5 with posterior fusion	15	7/0	7/0

Study of back and leg pain reduction between female versus male with t11 = −0.779, p-value = 0.226 for back pain and t11 = −1.431, p-value = 0.0901 for leg pain indicates that surgery was effective for both groups (female and male). None of the patients developed a spinous process fracture or needed reoperation. The blood loss was minimal, the length of the incisions was between 3.5 cm and 4 cm, and the operating time was between 45 minutes and 60 minutes. All patients were discharged on the day of the surgery. The follow-up is between 14 months and 24 months, with a median of 19 months.

## Discussion

The conventional decompressive laminectomy with removal of lamina and ligamentum flavum is a logical procedure to decompress a spinal canal that is narrowed due to thickened bone and ligament. This procedure is often supplemented with spinal fusion. Though this does bring relief to a large percentage of people it has certain disadvantages. It is an invasive approach that may not be suitable for older people. Furthermore, because it is an intraspinal procedure it can result in injury to the nerve roots, formation of scar tissue around nerve roots resulting in chronic pain syndrome, adjacent segment instability, epidural hematoma formation, and dural tear resulting in a spinal fluid leak. On the other hand, spinal distraction fixation systems are minimally invasive and the spinal canal or the capsular ligaments around the facets are not violated. Therefore, the chance for instability, spinal fluid leak, post-operative scarring with resulting pain, post-operative intraspinal clot formation, and injury to nerve root(s) is unlikely.

An interspinal distraction device can distract the interspinous space. This widens the foramen; widens the disc space resulting in a decrease in intradiscal pressure and disc bulge; straightens the interlaminar ligament which increases the diameter of the spinal canal, and decreases facet pain by decreasing pressure within the facet. Implanting this device is simple and minimally invasive with minimal risk for serious complications. Furthermore, since the procedure increases the height of the foramen and decreases intradiscal pressure, it reduces the risk of disc herniation at the treated level in the future. In addition, the procedure can be performed under general or local anesthesia, hospitalization is not required, and the patient can return to normal activity within a day or two. This system, therefore, gained some popularity in the last 10-15 years.

The early distractor systems were spacers. They were used as the only procedure for spinal stenosis or and in combination with a laminotomy. Though early results were very encouraging [3] some of the longer follow-up reports showed return of symptoms with a high rate of reoperation, radiculopathy, spinous process fracture, and new-onset radiculopathy [1,2].

The newer types listed in the introduction section included interspinal dynamic distraction fixation (ISDDF), which is a distraction stabilization with preservation of motion; and ISDRF which is a distraction stabilization system with rigid fixation.

In a cadaver study, the authors [13] observed that distraction opens the canal and foramen. In a cadaveric study [14], the results showed that for a single segment fusion there was no difference in stability of the fusion between pedicle screw fixation and interspinal fixation. In another cadaveric study, the authors concluded [15] that the ISDRF device fixates a spinal segment to enable immediate stability, distraction, decompression, and fusion. Furthermore, ISDRF devices produced effective interbody load and focal lordosis and perform well in comparison to bilateral pedicle screw fixation. In addition, incremental device manipulation showed predictable and safe trends regarding loading of the interbody space and spinous process. In a biomechanical study on seven cadaver spines [12], the authors concluded that interspinous distraction is not likely to cause adjacent level facet pain or accelerated facet joint degeneration. Furthermore, pain from the facet and annular bulge may be relieved.

The ISDDF device implants [4-6] have resulted in improvement of radicular and claudication pain, including facet joint pain. Furthermore, when they were compared with laminectomy and instrumented fusion for symptom relief, the results were equal. In addition, it maintained operative and adjacent level motion.

There are several reports on the ISDRF devices. Daentzer et al. [6] in a 10 patients series reported significant improvement in pain and disability using this device. After 24 months of follow-up, there was a statistically significant reduction in back and leg pain. There was also improvement in disability and range of motion. Nachanakanian et al. [8], in a series of 134 cases in which they implanted a similar device, concluded that this device is an efficient modality not only for adjacent segment disease but also for those who need rigid fixation. Sclafani et al. [10] used OsteoMed PrimaLOK SP Interspinous Fusion System (OsteoMed, Addison, TX), which is an ISDRF system in 53 patients. They had no complication, no reoperation, significant improvement in pain index score, and satisfactory McNab result in 48%. Postacchini et al. [9] used a Nuvasive ISDRF device in patients who underwent unilateral or bilateral decompressive laminotomy and observed a fusion rate of 84%. In the case report of 86 patients in the older age range with spinous process fixation and posterior fusion, the authors found that 91% had fusion with significant pain relief.

Goel et al. [16] reported a series of 70 patients who were treated with fusion alone using inter-articular screws. During the average follow-up period, 100% of patients had varying degrees of symptomatic relief with a very satisfactory satisfaction score. They concluded that “only fixation of the involved segment is necessary because spinal instability is the nodal point of the pathogenesis of spinal degeneration-related lumbar canal stenosis.”

According to Inspan LLC, the manufacturers of Inspan interspinous fixation device [17], biomechanical studies were performed on cadaver spine under the following conditions: (i) intact/control; (ii) Inspan only, (iii) Inspan with facet screws, (iv) Inspan with lateral interbody fixation, and (v) Inspan with lateral interbody fixation and facet screws. Each test consisted of 100 N of axial preload with ± 7.5 Nm of torque in flexion/extension, right/left lateral bending, and right/left axial rotation. The study showed that Inspan was biomechanically effective in increasing foraminal height and restricting flexion/extension motion. Furthermore, it was moderately effective in restricting axial or lateral bending. The wings of the system that are attached to the spinous process have strong spikes that are pushed down to the lamina and compressed down into the spinous processes. This drastically reduces the risk of the device slipping out. Furthermore, this is a screwless system. This reduces the risk of device breakdown.

In the current paper, the authors report 13 consecutive patients who had Inspan implants. All patients had a history of chronic pain, and seven patients has moderate to severe spinal stenosis. The operative time was between 45 minutes and 60 minutes. Statistically, there was a significant improvement in low back pain and lumbar radiculopathy pain. Though the subject number in this series is small the median for follow-up is 19 months and there is a statistically significant improvement in back and radicular leg pain. This procedure therefore in selected cases can negate the need for laminectomy and major spinal fusion surgery. 

## Conclusions

The Inspan interspinous distraction and stabilization system reported in this paper is a solid screwless fixation that works both as a distractor that decompresses the spinal canal and as a fusion device. It is a simple to use and safe system that eliminates the use of aggressive and invasive procedures such as pedicle screw and spinal cage type of fixation in the appropriate patient demographic. Though the number of test subjects is small, the follow-up timeframe is sufficiently long to showcase the effectiveness of ISP devices for back and leg pain reduction via decompression and stabilization. The results showing a statistically significant reduction in leg and back pain are encouraging. This procedure provides long-term benefit by leaving the level intact for future invasive procedures (should the need arises) like pedicle screw and cage type of spinal fusion. Furthermore, because it also involves posterior boney fusion, the effect should be good in the long term.
